# Modulation of cue-induced firing of ventral tegmental area dopamine neurons by leptin and ghrelin

**DOI:** 10.1038/ijo.2015.131

**Published:** 2015-08-11

**Authors:** G van der Plasse, R van Zessen, M C M Luijendijk, H Erkan, G D Stuber, G M J Ramakers, R A H Adan

**Affiliations:** 1 Department of Translational Neuroscience, Brain Center Rudolf Magnus, University Medical Center Utrecht, Utrecht, The Netherlands; 2 Master's Programme Neuroscience and Cognition, Utrecht University, Utrecht, The Netherlands; 3 Department of Psychiatry & Cell Biology and Physiology, UNC Neuroscience Center, University of North Carolina at Chapel Hill, Chapel Hill, NC, USA

## Abstract

**Background/objectives:**

The rewarding value of palatable foods contributes to overconsumption, even in satiated subjects. Midbrain dopaminergic activity in response to reward-predicting environmental stimuli drives reward-seeking and motivated behavior for food rewards. This mesolimbic dopamine (DA) system is sensitive to changes in energy balance, yet it has thus far not been established whether reward signaling of DA neurons *in vivo* is under control of hormones that signal appetite and energy balance such as ghrelin and leptin.

**Subjects/methods:**

We trained rats (*n*=11) on an operant task in which they could earn two different food rewards. We then implanted recording electrodes in the ventral tegmental area (VTA), and recorded from DA neurons during behavior. Subsequently, we assessed the effects of mild food restriction and pretreatment with the adipose tissue-derived anorexigenic hormone leptin or the orexigenic hormone ghrelin on VTA DA reward signaling.

**Results:**

Animals showed an increase in performance following mild food restriction (*P*=0.002). Importantly, food-cue induced DA firing increased when animals were food restricted (*P*=0.02), but was significantly attenuated after leptin pretreatment (*P*=0.00). While ghrelin did affect baseline DA activity (*P*=0.025), it did not affect cue-induced firing (*P*⩾0.353).

**Conclusions:**

Metabolic signals, such as leptin, affect food seeking, a process that is dependent on the formation of cue-reward outcomes and involves midbrain DA signaling. These data show that food restriction engages the encoding of food cues by VTA DA neurons at a millisecond level and leptin suppresses this activity. This suggests that leptin is a key in linking metabolic information to reward signaling.

## Introduction

The worldwide prevalence of obesity and the ongoing debate with respect to the existence of eating-addiction illustrates the importance of a precise understanding of the neurobiology of feeding behavior.^1^ The maintenance of a positive energy balance is regulated by multiple neural circuits that control energy expenditure and the procurement of energy sources. In addition to metabolic centers located in the hypothalamus that sense and regulate energy homeostasis,^2^ dopaminergic (DA) neurons in the midbrain (ventral tegmental area (VTA) and substantia nigra) have a crucial role in reinforcing food seeking behavior.^3, 4^

Activity of VTA DA neurons that project to the ventral striatum, which is important for feeding behavior,^5, 6^ is necessary for the formation of cue-reward associations and effort-related food seeking.^7^ Previous experiments in monkeys show that these DA neurons have an important role in signaling the value of food-predicting cues that drive motivated behavior.^8^ Importantly, this DA signal also encodes properties like reward identity and size, which allow for an accurate behavioral response following food availability.^9, 10, 11, 12^

Of particular interest for feeding behavior is that the activity of the midbrain DA system is modulated by metabolic state and feeding hormones.^13, 14, 15^ As such, under normal physiological conditions, the DA system can drive food-seeking behavior during hunger, and reduce this activity when satiated. Chronic food restriction, for example, reduces the levels of the anorexic hormone leptin levels^16^ and increases overall DA neuronal burst firing.^17, 18^ In contrast, suppression of dopaminergic activity is observed following administration of leptin in anesthetized rats.^19^ In direct opposition to leptin, the orexigenic hormone ghrelin increases DA release *in vivo*.^20^ Despite the correlation between feeding hormones, DA activity and metabolic state, the functional consequences of leptin and ghrelin on DA neuronal activity during behavior have not been established.

Previously reported effects observed after administration of these hormones show respectively decreased-, and increased motivation to obtain food reward.^21, 22^ Of particular relevance here are findings that show that leptin-deficient people show increased cue reactivity in DA-responsive brain regions such as the ventral striatum, even in conditions of satiation, and that leptin normalizes this activity.^23^ Similarly, several studies in human subjects indicate that leptin treatment of congenital-, and acquired leptin deficiency alters brain activity of reward-related brain areas such as the striatum and midbrain.^23, 24, 25^ Likewise, functional magnetic resonance imaging data show ghrelin to increase activity of areas that control appetitive behavior^26^ such as the mesolimbic system.^27^

These data suggest that leptin and ghrelin can modulate food seeking and activity of the reward circuitry. However, these studies do not identify the neuronal substrate through which these hormones act on the brain to alter cue responsivity.

Although leptin and ghrelin affect food seeking, it is unclear whether these hormones act directly on the signaling of reward-predicting stimuli. In this study, we determine the ability of ghrelin and leptin to affect the DA reward-system *during* reward-seeking behavior. These experiments show that a mild food restriction increases the activity of DA neurons in response to a reward-predicting cue, and pretreatment with leptin abolishes this activity. These data identify leptin as a key hormonal signal in the regulation of DA activity and establish a mechanism through which leptin signaling can modulate food seeking behavior.

## Materials and methods

All experiments were approved by the Animal Experimentation Committee of the University Utrecht and were carried out in agreement with Dutch Law (Wet op de Dierproeven, 1996) and European regulations (Guideline 86/609/EEC).

### Animals

Data were collected from 11 male Wistar rats (Harlan CPB, Horst, The Netherlands) weighing on average 391±12 g at the time of surgery. On arrival, the animals were individually housed, weighed and handled daily, and kept under a reversed day/night cycle (10:00–22:00 h lights off). Food and water were ad libitum available in the home-cage throughout the experiment.

### Apparatus

Animals were housed and measured in Perspex test chambers (50 × 50 × 35 cm) equipped with a house light, two trial lights and sound cues, three nosepoke units and three food-dispensers (Med Associates, Sandown Scientific, Middlesex, UK). Entries into the nosepoke unit and delivery of food pellets into the chamber were recorded through infra-red detection. Cues and feeders were controlled through a Med-pc interface that also registered all events. Additional event registration was achieved through coupling to the electrophysiology setup (see below).

### Surgery

Surgical procedures for the placement of recording electrodes were identical to those described earlier.^28^ In brief, after induction of anesthesia and exposure of the cranium, a hole was drilled over the VTA in the right hemisphere (5.6 mm posterior, 1.3 mm lateral to bregma).^29^ The dura was opened and electrodes were lowered into the brain under a 5° angle. Sixteen electrodes were bundled into four tetrodes that were mounted in a Harlan 4 Drive (Neuralynx, Bozeman, MT, USA). After lowering the tetrodes ~4 mm into the brain the drive was anchored to skull-screws with dental cement. Following surgery, tetrodes were further lowered into the target area (~8.2 mm DV). Subsequently, the animals were given analgesics for 2 days, and at least 7 days of recovery before electrophysiological recordings started.

### Recording

Recordings were performed in a test chamber that was identical to the home-cage. Data were recorded at 40 Khz using a Multichannel Acquisition Processor (MAP) recording system (Plexon, Dallas, TX, USA), as described earlier.^28^ A 750-μs data sample was stored whenever the signal crossed a preset voltage threshold. Data were clustered and analyzed offline (see below).

### Behavioral procedure

#### Behavioral task

**Figure 1:**
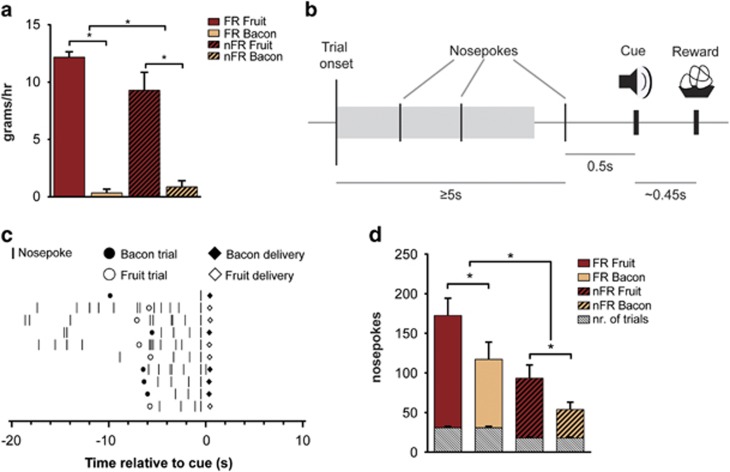
Reward preference, task design and performance. (**a**) Total intake (g) of fruit- and bacon-flavored rewards during 1-hour free-access in food restricted (FR) and non-restricted (nFR) animals. Data indicate preference for fruit over bacon pellets and increased intake following food restriction. (**b**) Flowchart of the behavioral task. At trial onset a tone signals the availability of a bacon or fruit reward. The first nosepoke following a 5-s interval triggers a sound cue (0.5 s following the nosepoke) and reward delivery (~0.45 s later). (**c**) Nosepoke activity of a single animal during the first 10 trials of a single session. Rasters are zeroed on the cue. (**d**) Nosepokes during task execution. Significantly more nosepokes are made for fruit- than for bacon-flavored pellets, food restriction furthermore increases total number of nosepokes. The average number of trials per reward for each session type is depicted in gray. *Significant difference at *P*<0.05. All data are presented as mean±s.e.m.

After arrival, animals were trained daily in their home cage on a fixed-ratio 1 task (1 h). During this behavioral task, a single nosepoke led to the delivery of a foodpellet. After animals learned to associate the nosepoke with a food reward, they were trained on the main paradigm. Task onset was signaled by a houselight, which remained illuminated for the duration of the test session (1 h). Following a 30- to 45-s intertrial interval, trial onset was signaled by onset of the light in the nosepoke hole and a sound cue (Figure 1b). Each reward type (see below) was presented in a pseudo-random manner and was signaled by a unique tone and light cue (Figures 1b and c). To obtain the reward, animals had to nosepoke at one of two reward sites (corresponding to the available reward type). The first nosepoke after a 5-s interval led to the onset of a feeder and delivery of the food reward. The sound of this feeder onset served as a proximal sound cue, and was used for most of the electrophysiological analyses. Food delivery was approximately 450 ms after feeder activation, followed by the cessation of the sound/light cue presentation and trial termination. After completion of the trial, the intertrial interval was reset. During daily training sessions, water and food were freely available. To assess the effect of reward preference on task execution and reward processing, two rewards that were differentially preferred were used. These rewards were bacon-flavored and fruit-flavored 190 mg isocaloric pellets (resp. 3.37 and 3.45 kcal g^−1^; Bio Serv). ‘Bacon' and ‘fruit' trials were presented in a pseudo-random order to prevent specific anticipatory activity prior to stimulus presentation (4-trial blocks).

#### Food preference task

Reward preference was assessed in the recording chamber on separate test days and all experimental conditions were tested (vehicle, hormone and food restriction). Unlimited access to both rewards was given for a period of 1 h. Before and after the session, the total amount of food was weighed and intake was calculated.

#### Experimental procedure

At the start of an experimental day (~11:30 h; 1.5 h into the dark phase), an animal was placed in the recording chamber with either ad libitum or no access to food (food restriction). During this period, ad-lib fed rats have an average intake of 3.34±1.6 g. Two hours later, the animal was injected intraperitoneal (i.p.) with hormone (leptin or ghrelin) or vehicle (saline) and connected to the recording device. After a 5-min interval, the behavioral task and the electrophysiological recording started. Following task execution, the animals remained in the recording chamber to record baseline neuronal activity (30 min), and neuronal activity following administration of apomorphine (i.p.). To ensure that new neurons were recorded in each condition, the tetrodes were lowered at least 40 μm between sessions. Animals were pseudo-randomly chosen for each recording session such that no animal was measured on two consecutive days.

#### Pharmacological intervention

Ghrelin (250 μg kg^−1^; Tocris, Bristol, UK), leptin (1 mg/kg; National Hormone and Peptide Program (NHPP), USA) as well as vehicle (saline) were injected i.p. 5 min before recordings started. These concentration were previously shown to affect food intake and neuronal activity.^28^ Saline was injected in an identical volume (0.1 ml per 100 g) to control for neuronal responses to the injection. Apomorphine (0.1 mg kg^−1^; Sigma-Aldrich, Saint Louis, MO, USA) was administered i.p. 30 min following task execution, to identify DA neurons.

#### Data analysis

All statistical analyses were performed using SPSS for Windows (IBM; Armonk, NY, USA) unless stated otherwise. Equality of variance of the differences between groups was assessed using Mauchly's test of sphericity. In case variances were not equal, the degrees of freedom were adjusted by means of a Huyn-Feldt correction to reduce the chance of type-one errors.

#### Behavior

For the behavioral performance during recording sessions the following measures were analyzed; total number of trials and total number of nosepokes per reward and response latency. During the free-access preference test, the total consumption of each reward was measured.

#### Electrophysiology

Single neurons were isolated by offline cluster procedures (Offline Sorter x64 V3; Plexon). Cells with a baseline firing frequency of less than 0.1 Hz over the whole recording were not considered for analysis (identical to Van der Plasse *et al.*^28^). Neurons were classified as DA neurons based on their response to the dopamine 2 receptor agonist apomorphine (⩾20% reduction in firing frequency with respect to pre-treatment activity).^30^

To exclude effects of the injection procedure on neuronal activity, data from 30 s before and until 30 s after injections were not included in the analyses. Treatment-induced changes in baseline firing frequency were analyzed with a one-way ANOVA and *post hoc* *t*-test. Burst activity was quantified by computing the fraction of total spikes that occurred within a burst, in accordance with earlier used burst criteria.^30, 31^

#### Event-related firing

Population analyses of neuronal activity were performed to assess neuronal response to the task-related events, cue light (that is, trial onset and type identifier), reward cue (that is, feeder sound), and reward presentation. Reward presentation was measured by an infrared detector placed to detect food pellets at the moment they fell into the cage.

For each single unit, peri-event time histograms were constructed (Neuroexplorer V4, NEX Technologies, Madison, AL, USA) per event. Subsequently, binned data (100 ms) were exported and normalized to baseline using Z-scores based on average firing activity during 10 s of activity in the intertrial interval. Averaged responses to events were then calculated per condition (hormone/restriction) and neuron type (DA/other). Statistical analyses were performed by means of a repeated-measures ANOVA and *post hoc* *t*-tests.

### Histology

After the last recording session, rats were anesthetized and the final position of the tetrodes was marked by passing a 10-s, 25-μA current through each tetrode. After 24 h, the animals were killed, and the brains were removed and stored overnight in paraformaldehyde. Subsequently, brains were transferred to a 30% sucrose solution with 0.05% sodium azide. Brain sections (40 μm) were cut using a vibratome and Nissl stained to identify the location of the final position of the tetrodes.

## Results

### Task performance and free choice access reflects reward preference

Animals were trained on a behavioral task in which two different food rewards (fruit- and bacon flavored pellets) could be obtained following a nosepoke response (Figures 1b and c). During a 1-h free access to both reward types all animals showed preference for fruit-flavored pellets over bacon-flavored rewards (Figure 1a, *t*⩾5.065, *P*=0.000). Furthermore, multivariate analysis of the effects of food restriction on nosepokes per reward type showed a significant effect (F(2,14)=5,573, *P*=0.017). Subsequent analysis showed that the number of nosepokes for fruit-flavored rewards was significantly greater than for bacon-flavored rewards under conditions of both food restriction and non-food restriction (resp. F (1,15)=6,369, *P*=0.023; F (1,15)=7,781, *P*=0.014; Figure 1d).

Although food restriction in these animals significantly increased overall intake during the 1-h free access (*t*=1.839, *P*=0.046), it did not affect preference (Figure 1a). Increased intake following restriction was also reflected in behavioral performance. Animals made significantly more nosepokes under conditions of food restriction than when food was freely available (Figure 1d; *t*=3.377, *P*=0.002). In addition, food restriction (that is, 2 h of restriction) significantly increased the number of trials that animals performed (30.8±1.8) compared with free-fed animals (18±1.1 trials; *t*=4.392, *P*=0.001; Figure 1d, gray bars). Under ad lib conditions, when food was available during testing, no chow was consumed. Pre-treatment with ghrelin or leptin did not affect the number or delay of nosepokes compared with saline control (resp. F=0.812, *P*=0.462 and F=0.106, *P*=0.900), nor reward preference (F=1.597, *P*=0.243), allowing for the analysis of reward processing independent of task execution.

These data thus indicate that rats show clear reward preference and increased reward-seeking behavior after mild food restriction. Moreover, under these conditions leptin nor ghrelin affected task performance.

### Measurement of midbrain neuronal activity revealed neuron type-dependent firing in response to cue presentation

**Figure 2:**
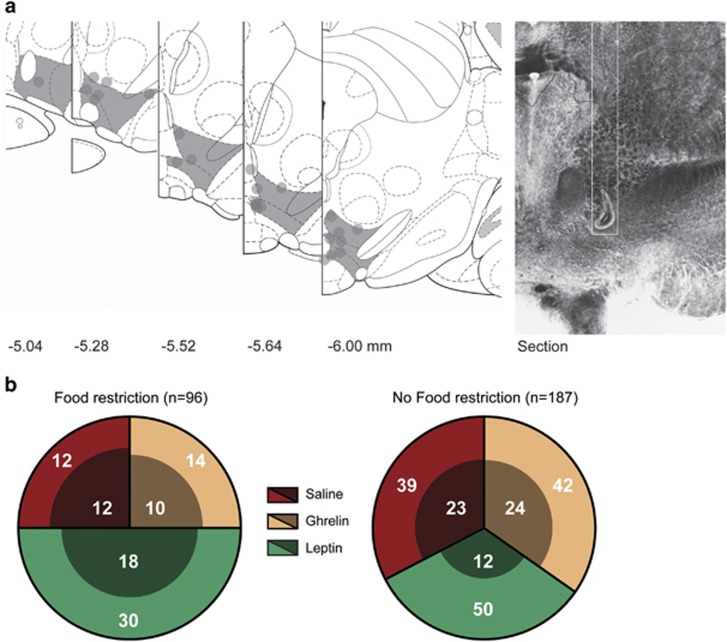
(**a**) Recording sites in the midbrain. (left) Shaded areas delineate the ventral tegmental area (VTA), tetrode end points are marked as circles. Relative distance to bregma is indicated below. (right) Photograph of a histological section showing a tetrode track (outlined in white) and end point. (**b**) Overview of the number of recorded neurons per session and condition. Inner circles show dopamine (DA) neurons and non-DA neurons are shown in the outer circle.

To investigate the effect of short food restriction (2 h) and hormone treatment on reward processing of midbrain neurons, we recorded single-cell neuronal activity in the VTA of rats (*n*=11) performing an operant task to receive two different food rewards. Before performing this task, animals were pretreated with ghrelin, leptin or vehicle (saline). During these experiments, 283 midbrain neurons were measured (Figures 2a and b). Of these, 99 were classified as putative DA (Figure 2b, inner circle) based on their inhibition by the dopamine 2 receptor agonist apomorphine.To rule out effects of apomorphine on task performance, we administered the hormone 30 min after the end of the behavioral task. The remaining neuronal population was uniformly categorized as non-DA. Figure 2b shows a complete overview of the number of neurons measured, per condition.

We first assessed the effects of hormone treatment and food restriction on baseline firing frequency (Table 1). ANOVA analyses revealed that hormone treatment affected firing activity under conditions of food restriction (F=5.100, *P*=0.011), but not when animals were fed ad lib (F=0.328, *P*=0.722). In line with previous findings from anesthetized animals,^19^ subsequent *post hoc* analyses showed that leptin reduces firing frequency of DA neurons in awake rats, during food restriction (F=5.761, *P*=0.023). Unexpectedly, a similar reduction of hormone-induced neuronal activity was found for ghrelin under these conditions (F=5.862, *P*=0.025; see Abizaid *et al.*^32^). Burst-firing analysis of DA neurons revealed that under saline conditions there was no difference in the fraction of spikes that occur within a burst between restriction and non-food restriction (21–23%, burst percentage is similar to earlier reports^30, 31^). Under conditions of food restriction, burst firing was significantly decreased following ghrelin pre-treatment (F=8.833, *P*=0.008) and unaffected by leptin. Thus, both ghrelin and leptin decrease the baseline firing frequency of DA neurons, but only during a mild food restriction.

**Figure 3:**
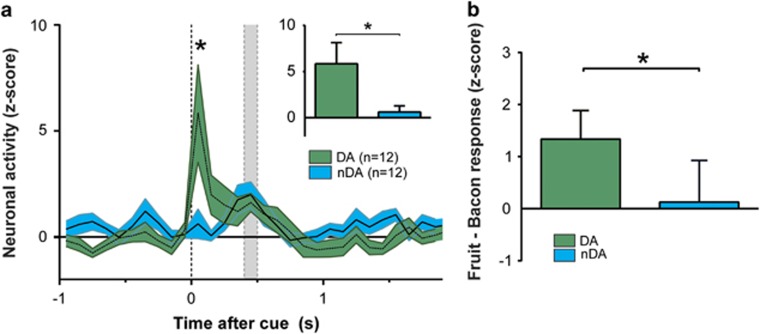
Electrophysiological recordings in the rat midbrain. (**a**) Significant cue-induced neuronal firing in dopamine (DA)-, but not in non-DA (nDA) neurons under food-restriction conditions (resp. green and blue trace). The insert shows a significant difference between both neuron types after cue presentation (first 100 ms). (**b**) Neuronal activity in response to presentation fruit- and bacon-flavored rewards in putative dopamine (green) and non-dopamine neurons (blue), 300–400 ms after cue onset. All data represented as normalized z-scores. *Significant difference at *P*<0.05. Shaded gray area represents the reward-delivery interval.

In accordance with the known neurophysiological properties of midbrain DA neurons, under conditions of a mild food restriction DA neurons showed clear increases in activity in response to a cue presentation that predicted the upcoming reward. Whereas these neurons showed a clear cue-induced increase in firing activity, non-DA neurons showed no such response (Figure 2a). A repeated-measures ANOVA comparison between cue-induced firing in DA and non-DA neurons revealed a significant effect of both time (F=4.412, *P=*0.001), and group × time (F=3.125, *P*=0.009). Figure 3a shows the average cue-induced changes for both cell types (*t*-test; 3.524, *P*=0.001).

Also, analysis of the whole population revealed a significant increase in neuronal activity following cue onset with respect to baseline (rmANOVA, F=4.039, *P*=0.004; simple-first contrast; F=5.562, *P*=0.02). Furthermore, actual *reward delivery* did not induce differential firing in DA and non-DA neurons (F=0.313, *P*=0.582), indicating that activity in DA neurons is not attributable to the sensory processing of the actual reward.

As DA neurons have previously been shown to encode relative reward value, and in these experiments rats showed preference for fruit-flavored rewards, we assessed whether DA neurons exhibit differential firing in response to cue and reward presentation. In an unbiased approach we compared the first 500 ms after cue onset, for all neurons recorded under saline conditions. We found differential neuronal activity between the two rewards 300–400 ms after cue onset with increased activity for the preferred fruit reward, similar to what has been reported.^33^ Importantly, this difference was significant only in the population of DA neurons, but *not* in other recorded neurons (Figure 3b). These results thus show class-specific neuronal activity that corroborate known properties of DA neurons.

### Food restriction is necessary for cue-induced firing of DA neurons

To assess the influence of feeding on reward processing, we measured midbrain VTA neuronal activity under conditions of short food restriction or ad lib access to chow.

**Figure 4:**
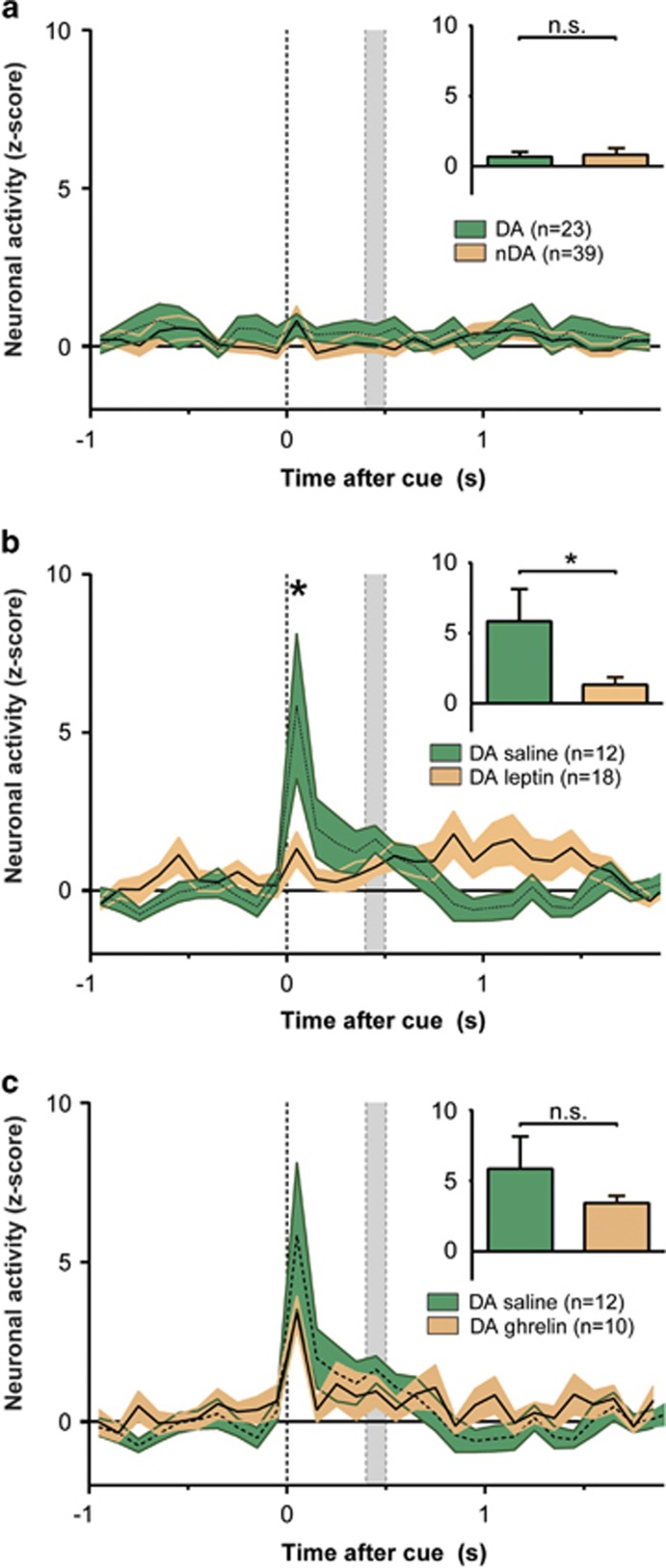
Leptin and access to food attenuates cue-induced firing. (**a**) The absence of cue-induced firing in dopamine (DA) and non-DA (nDA) neurons (resp. green and orange trace) following ad lib access to food. No significant difference was found between both neuron types after cue presentation (first 100 ms; insert). (**b**) Leptin (orange trace) attenuates cue-induced firing of DA neurons in food-restricted animals (green trace). The insert shows a significant difference between both treatments after cue presentation (first 100 ms), the control group is identical to the group shown in Figure 3a. (**c**) Ghrelin does not affect cue-induced firing in food-restricted animals (orange trace) compared with saline-treated animals (green trace). The insert shows the average firing activity for both conditions after cue presentation (first 100 ms), the control group is identical to the group shown in Figure 3a. All data represented as normalized z-scores. *Significant difference *P*<0.05. Shaded gray area represents the reward-delivery interval.

Surprisingly, when measured under ad lib-fed conditions neither DA nor non-DA neurons show increased firing activity in response to cue or reward presentation (Figure 4a). Analysis of variance (repeated-measures ANOVA) revealed that there was no effect of time, group or interaction (F>0.592, *P*>0.378) in saline-treated animals. Similarly, when all neurons were included in the analysis, no significant effect of time was observed (F=1.010, *P*=0.426). Together, these data thus indicate that under ad libitum access to food and water, DA neurons show minimal responsivity to reward predicting cues.

### Pre-treatment with the anorexic hormone leptin suppresses food restriction-induced firing of DA neurons

To examine the effect of feeding hormones on cue-induced activity of DA neurons, we analyzed whether administration of ghrelin and leptin modulated this activity.

Leptin administration before recording sessions significantly decreased cue-induced neuronal activity in DA neurons measured under conditions of food-restriction. A repeated-measures comparison between saline and leptin-pretreatment conditions revealed a significant effect of time (Figure 4b, F=4.076, *P*=0.001) and a group × time interaction (F=3.467, *P*=0.002). *Post hoc* analysis furthermore indicated a significant difference between groups following cue presentation (*t*=4.233, *P*=0.000). In contrast, ghrelin treatment did not affect cue responding compared with saline-treated animals (Figure 4c, F⩽1.123, *P*⩾0.353). In ad lib-fed animals leptin or ghrelin administration did not alter event-related firing of DA neurons.

Overall we find that activity of midbrain DA neurons is increased during proximal cue presentation, in accordance with earlier findings.^8^ Importantly, this transient increase in event-related firing is only observed in animals that were food restricted and this increase is abolished by administration of leptin but not affected by ghrelin.

## Discussion

Here we show that a cue that predicts the availability of a food reward induces DA firing, but only in conditions of food restriction. Moreover, the anorexigenic hormone leptin can suppress this cue-induced neuronal activity. As such, these data provide a neural substrate through which metabolic information modulates encoding of cues associated with food reward on a millisecond timescale by DA neurons.

Neuronal activity measured in mildly fasted rats (that is, 2 h food restriction) revealed that while midbrain DA neurons strongly increase their activity to reward-predicting cues, both leptin pre-treatment and free access to chow before task execution abolished this cue-induced firing. Although ghrelin did affect baseline neuronal activity, no evidence was found for modulation of task-related activity of DA neurons.

Of particular relevance for these data is earlier work that showed that under anesthesia VTA DA neurons decrease their firing to intravenous leptin, but the functional consequences of leptin on DA signaling during behavior remained unclear.^19^ Importantly, as leptin affected neither reward preference, number of nosepokes, nor latency to nosepoke, these data suggest that leptin acts directly on the signaling of reward information. The subset of neurons that we recorded from under these conditions thus shows sensitivity to food restriction and to leptin but surprisingly did not significantly decrease task performance.

It is well established that operant behavior that requires a low motivational effort, like the single nosepoke that was required in this task, is resistant to accumbal DA lesions,^7, 34, 35^ similar to feeding behavior in a setting where food is freely available.^36^ Importantly, cue-induced accumbal DA release is seen during these tasks.^3^ This suggests that DA neuronal activity is present, but not required for basic task execution on a low-effort paradigm. Possibly DA activity, reflecting reward-prediction errors, affects task performance on a longer timescale. It is thus likely that the behavioral performance on this task is largely mediated by brain activity outside the VTA. Importantly, while leptin effects on behavior are seen on high motivational tasks like progressive ratio tasks,^21^ they are most pronounced on low effort feeding after multiple injections, or over multiple hours.^37, 38^ Therefore, the elegance of this study is that we provide a task that is low in motivational load, but still induces DA burst firing. The reduction in firing we see by leptin therefore does not modify task performance, ruling out the possible confound that leptins effect on behavior is the cause for the changes in DA firing that we observe.

Systemic treatment with the orexigenic hormone ghrelin decreased overall baseline activity of DA neurons under conditions of food restriction, but did not affect cue-induced activity. Such a dissociation between basal activity and burst firing has been reported before. Hyland *et al.*, for instance showed that the average firing activity is not different between high and low bursting groups of neurons.^30^ In addition, modulation of excitatory inputs to VTA DA neurons affects burst firing, but not basal activity.^39^

Different effects of ghrelin on DA activity have been reported. Whereas in human subjects administration of ghrelin has been shown to increase activity in the midbrain, among other areas (for example, Malik *et al.*^26^), reports from rodent studies are less consistent. Cone *et al.*^40^ previously showed ventricular, but not VTA, ghrelin injections increase accumbal DA release. Jerlhag *et al.*^20^ found that intra-VTA ghrelin injections increase accumbal DA levels. These seemingly contradictory results are possibly due to the different routes of administration. As ghrelin receptors are expressed throughout brain and periphery,^41^ we chose the route of endogenous ghrelin by i.p. injection.

The recorded DA neurons show differential firing between a preferred and non-preferred reward, in line with earlier reports,^9, 10, 11, 12^ and show burst patterns similar to those of DA neurons reported before.^30, 31^ Although these data corroborate earlier work on the function of DA in the signaling of reward information, a need for (mild) food restriction to induce cue-induced firing has not been reported before. It should be noted that the majority of earlier work on DA signaling was done under conditions of food (or water) restriction.^30, 42, 43^ It has long been established that food restriction increases general activity and motivation, as well as the rewarding effect of lateral hypothalamic self-stimulation.^15, 44^ More recently, it was found that food restriction changed basal activity of DA neurons,^18^ and here for the first time we show that it enhances reward processing by midbrain DA neurons.

The lateral hypothalamus sends dense projections to the VTA, containing a wide range of neuropeptides that modulate feeding behavior, including orexin/hypocretin, melanin-concentrating hormone and neurotensin. Interestingly, it has been reported that a subset of LH neurons that contain the leptin receptor project directly to the VTA, and might modulate DA activity.^45^ Although these LH leptin receptor containing neurons are presumed GABAergic,^46^ it has also been shown that leptin decreases glutamate-mediated excitatory inputs to DA neurons.^47^ Furthermore, receptors for leptin are also expressed on VTA DA neurons, enabling the possibility of a direct modulation.^48^ Taken together, it is likely that leptins effect on different brain structures contribute to downstream changes in DA signaling. Although we show that leptin and short food restriction modulate cue-induced firing of DA neurons, it remains to be investigated whether this is through a direct activation of receptors in the VTA, or whether inputs from other brain areas, like the LH, are necessary for these effects.

DA neurons respond to reward-predicting cues with a transient increase in firing activity, enabling signaling of a reward-prediction error.^8^ Thus far, no studies have directly assessed the effects of feeding hormones on event-related firing of DA neurons in animals that are actively engaged in a reward-seeking task. The present data show that food restriction and leptin modulate DA signaling to cues associated with a food reward, without affecting task performance or actual feeding itself. Specifically, these data suggest that decreased reward value, through leptin administration, decreases cue-induced firing of midbrain DA neurons. In a similar manner, pre-feeding decreases neuronal responses to such reward-prediction cues. These findings confirm and extent previous reports on the effects of metabolic state and leptin treatment on the processing of food cues in humans. In these, leptin affected the response of reward-related neural structures upon presentation of food pictures without actual consumption.^23, 24, 25^ However, a direct modulation of DA activity by leptin, on a millisecond time-scale, was not shown before.

As such, these data provide the first evidence for a direct effect of metabolic signaling on the processing of reward-related information by midbrain DA neurons and provide a neuronal substrate of satiety-induced changes in motivated behavior. These data bring a new perspective to how leptin affects the midbrain DA system which is relevant to eating disorders and obesity.

## Figures and Tables

**Table 1:** Baseline firing frequencies and burst activity per condition

	*No food restriction*		*Food restriction*	
	*Firing freq.*	*SWB*	*Firing freq.*	*SWB*
Saline	1.23 (0.41)	0.21 (0.06)	1.03 (0.26)	0.23 (0.04)
Leptin	0.73 (0.27)	0.19 (0.09)	0.44 (0.1)*	0.32 (0.06)
Ghrelin	1.58 (0.87)	0.21 (0.06)	0.31 (0.24)*	0.08 (0.2)**

Overview of average firing frequency (±s.e.m.), and the fraction of spikes that occur within a burst (SWB; ±s.e.m.) of putative dopamine (DA) neurons. *Significant difference *P*<0.05 and **Significant difference *P*<0.01.
